# Induced pluripotent stem cell‐conditional medium inhibits H9C2 cardiomyocytes apoptosis via autophagy flux and Wnt/β‐catenin pathway

**DOI:** 10.1111/jcmm.14327

**Published:** 2019-04-07

**Authors:** Xiaoling Guo, Xiaohong Gu, Sohun Hareshwaree, Xing Rong, Lei Li, Maoping Chu

**Affiliations:** ^1^ Center of Scientific Research The Second Affiliated Hospital and Yuying Children's Hospital of Wenzhou Medical University Wenzhou China; ^2^ Institute of Cardiovascular Development and Translational Medicine The Second Affiliated Hospital and Yuying Children's Hospital of Wenzhou Medical University Wenzhou China

**Keywords:** apoptosis, H9C2 cardiomyocytes, induced pluripotent stem cell‐derived conditioned medium, myocardial infarction, proliferation

## Abstract

Induced pluripotent stem cell‐derived conditioned medium (iPS‐CM) could improve cell viability in many types of cells and may be a better alternative for the treatment of myocardial infarction. This study aimed to examine the influence of iPS‐CM on anti‐apoptosis and the proliferation of H9C2 cardiomyocytes and investigate the underlying mechanisms. H9C2 cardiomyocytes were exposed to 200 μmol/L hydrogen peroxide (H_2_O_2_) for 24 hours with or without pre‐treatment with iPS‐CM. The ratio of apoptotic cells, the loss of mitochondrial membrane potential (△Ψm) and the levels of intracellular reactive oxygen species were analysed by flow cytometric analysis. The expression levels of BCL‐2 and BAX proteins were analysed by Western blot. Cell proliferation was assessed using cell cycle and EdU staining assays. To study cell senescence, senescence‐associated β‐galactosidase (SA‐β‐gal) staining was conducted. The levels of malondialdehyde, superoxide dismutase and glutathione were also quantified using commercially available enzymatic kits. The results showed that iPS‐CM containing basic fibroblast growth factor significantly reduced H_2_O_2_‐induced H9C2 cardiomyocyte apoptosis by activating the autophagy flux pathway, promoted cardiomyocyte proliferation by up‐regulating the Wnt/β‐catenin pathway and inhibited oxidative stress and cell senescence. In conclusion, iPS‐CM effectively enhanced the cell viability of H9C2 cardiomyocytes and could potentially be used to inhibit cardiomyocytes apoptosis to treat myocardial infarction in the future.

## 1 INTRODUCTION

In recent years, myocardial infarction has become one of the leading causes of mortality worldwide.^1^ Myocardial infarction is defined as necrosis of cardiomyocytes as a result of prolonged ischaemia, which is responsible for heart failure, cardiac fibrosis and sudden death.^2^, ^3^ Oxidative stress plays an important role in cardiomyocyte proliferation, apoptosis and differentiation.^4^ Currently, stem cell therapies for myocardial infarction have become promising alternatives to repair and regenerate injured tissues, and their applications are being extensively studied in various diseases.^5^, ^6^, ^7^, ^8^, ^9^, ^10^, ^11^

Induced pluripotent stem cells (iPSCs), which can be directly obtained from adult cells using reprogramming factors (Oct 3/4, Sox2, Klf4 and c‐Myc),^12^ resemble embryonic stem cells with the abilities of self‐renewal and differentiation into the three germ layers.^13^ However, iPSCs have no immune rejection and ethical issues, which offer an attractive platform for disease model, pharmaceutical screening, and so on.^14^, ^15^ So far, iPSCs have been successfully differentiated into cardiomyocytes.^16^, ^17^ and can achieve in situ regeneration in infracted adult mouse hearts.^18^ However, there are risks of tumorigenesis in rat hearts after cell transplantation.^19^, ^20^

Conditioned medium could affect cell functions and viability via biologically active components. It has been reported that basic fibroblast growth factor (bFGF), nerve growth factor, hepatocyte growth factor, vascular endothelial growth factor (VEGF), insulin‐like growth factor (IGF‐1) and brain‐derived neurotrophic factor could be secreted in the supernatant of cultured stem cells, which could improve cell viability.^21^, ^22^, ^23^ Neel and Singla reported that iPS‐derived conditioned medium (iPS‐CM) could reduce the occurrence of cardiac apoptotic nuclei in a diabetic cardiomyopathy rat model.^8^ Lian et al showed that iPS‐CM improved the proliferation and anti‐apoptotic abilities of human adipose‐derived stem cells.^24^ Zhang et al concluded that paracrine factors of iPSCs could inhibit stress‐induced senescence of H9C2 cardiomyocytes by inhibiting the p53‐p21 and p16‐pRb pathways.^25^ The stimulation of cell proliferation and anti‐apoptosis caused by iPS‐CM is mainly because of the secreted cytokines that do not cause tumour formation.^8^, ^24^, ^25^, ^26^, ^27^ There is the report that showed iPSCs and iPS‐CM have similar therapeutic effects on lung injury through the same signalling pathway.^26^ Therefore, iPS‐CM could be used to treat myocardial infarction. However, it is imperative to understand how and which factors of iPS‐CM affect cardiomyocyte viability and functions prior to of myocardial infarction therapy.

In this study, we investigated the effects of iPS‐CM on the proliferation and H_2_O_2_‐induced apoptosis of H9C2 cardiomyocytes and evaluated the influences of iPS‐CM on cell senescence and oxidative stress. Moreover, the potential mechanisms underlying the effects of iPS‐CM on H9C2 cardiomyocytes were explored. This study aimed to develop an effective method to improve the activities of cardiomyocytes and offered a potential therapeutic approach for myocardial infarction.

## 2 MATERIALS AND METHODS

### 2.1 Materials

H9C2 cardiomyocytes were obtained from the Chinese Academy of Sciences (Beijing, China). Hydrogen peroxide was purchased from Sigma‐Aldrich (MO, USA). Matrigel was purchased from BD Bioscience (NJ, USA). Low‐glucose Dulbecco's Modified Eagle's Medium, foetal bovine serum (FBS), 1% penicillin and streptomycin and 0.05% trypsin‐EDTA were purchased from Gibco (NY, USA). mTeSR1 medium was purchased from StemCell Technologies (BC, Canada). Cell counting kit‐8 (CCK‐8) was bought from BestBio (Shanghai, China). An ABI Prism 5, 59, 6, 69‐tetrachloro‐1, 19, 3, 39‐tetraethylbenzimi‐dazolylcarbocyanine iodide (JC‐1) assay kit, cell cycle kit and Annexin V‐FITC/propidium iodide (PI) apoptosis detection kit were purchased from KeyGEN (Nanjing, China). The fluorescent dye 2′7′‐dichlorofluorescin diacetate assay kit was obtained from Qcbio Science & Technologies (Shanghai, China). An enzyme‐linked immunosorbent assay (ELISA) kit was obtained from Chemicon International (CA, USA). Malondialdehyde (MDA), superoxide dismutase (SOD) and glutathione (GSH) were measured using assay kits from Jiancheng Biochemical Inc. (Nanjing, China). SA‐β‐gal staining solution was purchased from the Beyotime Institute of Biotechnology (Nantong, China). The in situ Cell Death Detection kit was purchased from Roche (Basel, Switzerland).

### 2.2 Cell culture

H9C2 cardiomyocytes were cultured in medium (DMEM‐HG) containing High‐Glucose Dulbecco's Modified Eagle's Medium, 10% FBS, and 1% penicillin and streptomycin. The culture conditions were maintained at 37°C in a 5% CO_2_ incubator. The cells were subcultured at confluence.

### 2.3 Preparation of iPS‐CM

Human iPSCs were acquired from the Guangzhou Institute of Biomedicine and Health, Chinese Academy of Sciences. iPSCs were produced from the umbilical cord matrix and amniotic mesenchymal cells through the transduction of the retroviral factors Oct4, Sox2, c‐Myc and Klf4.^28^ Previously, established protocols were followed to culture the iPSCs.^29^ Briefly, culture dishes were coated with 1% Matrigel at least 30 minutes before cell seeding. Then the iPSCs were cultured in mTeSR1 medium in a 37°C incubator with 5% CO_2_. Every 6 days, the iPSCs were subcultured using 0.05% trypsin‐EDTA at 37°C for 5 minutes, and then seeded onto 1% Matrigel‐coated culture plates. The supernatant of iPSCs was derived from the mTeSR1 medium cultured with iPSCs for 1 day. The supernatant was filtered (using a 0.22 μm filter) to remove dead cells and cell debris. The supernatant was then stored at −80°C for at least 2 weeks. The iPSC supernatant was mixed with DMEM‐HG at a ratio of 1:2 to obtain iPS‐CM.

### 2.4 CCK‐8 assay

Cell counting kit‐8 was used to measure cell viability under different conditions. Briefly, H9C2 cardiomyocytes (1 × 10^3^ cells/well) were seeded into 96‐well plates. The cells were then treated under different conditions and incubated for 24 hours at 37°C in a 5% CO_2_ incubator. Subsequently, 10 μL of CCK‐8 solution was added to each well and incubated for 4 hours, after which the absorbance at 490 nm was measured using a microplate reader (Thermo, MA, USA).

### 2.5 Apoptosis model establishment

H9C2 cardiomyocytes were seeded into 6‐well plates (1 × 10^6^ cells/well). The cells were incubated at 37°C in a 5% CO_2_ incubator for 24 hours. Different concentrations of H_2_O_2_ (0, 50, 100, 150, 200, 250, 300 and 350 μmol/L) were added to each well and then incubated for 24 hours. Bright field microscopy was conducted using an inverted fluorescence microscope (Nikon, Tokyo, Japan).

### 2.6 TUNEL staining

The TUNEL assay was conducted to detect cell apoptosis using an in situ Cell Death Detection kit according to the manufacturer's instructions. Briefly, H9C2 cardiomyocytes with different concentrations of H_2_O_2_ treatments were fixed with 4% paraformaldehyde for 1 hour at room temperature and washed with PBS three times for 5 minutes each wash. Then, cells were incubated with blocking solution (3% H_2_O_2_ in methanol) for 10 minutes at room temperature and washed with PBS three times for 5 minutes each wash. Then, cells were incubated in permeabilisation solution (0.1% Triton X‐100) for 2 minutes on ice and treated with TdT/dUTP FITC labelling reaction mixture for 1 hour at 37°C in the dark, followed by three rinses with PBS. Cell nuclei were positive if they were labelled with FITC (green), whereas DAPI staining indicated the cell nucleus under the florescence microscope. The positive staining cells were counted using Image‐Pro Plus 6.0 software.

### 2.7 Annexin V and PI assay

Annexin V and PI assays were conducted to measure the apoptosis of H9C2 cardiomyocytes treated with H_2_O_2_. H9C2 cardiomyocytes were seeded into 6‐well plates (1 × 10^6^ cells/well). The cells were then allowed to grow in DMEM‐HG and iPS‐CM for 24 hours at 37°C in a 5% CO_2_ incubator. Then, 200 μmol/L of H_2_O_2_ was added to each medium for 24 hours to induce apoptosis, while there was another group that received 30 ng/mL exogenous bFGF together with H_2_O_2_ as the DMEM‐HG+bFGF+H_2_O_2_ group. Then, H9C2 cardiomyocytes were collected and washed with PBS. The cells were then resuspended in 200 μL of Annexin V‐binding buffer and stained with 5 μL of FITC‐labelled Annexin V and 5 μL of PI for 20 minutes. The stained cells were then analysed by flow cytometry.

### 2.8 Measurement of mitochondrial membrane potential (Δψm)

The mitochondrial membrane potential (△Ψm) was measured using a 5, 59, 6, 69‐tetrachloro‐1, 19, 3, 39‐tetraethylbenzimi‐dazolylcarbocyanine iodide (JC‐1) assay kit. The H9C2 cardiomyocytes (1 × 10^6^ cells/well) were plated into 6‐well plates and cultured with DMEM‐HG and iPS‐CM at 37°C in a 5% CO_2_ incubator for 24 hours. Then, the cells were treated with 200 μmol/L H_2_O_2_ for 24 hours to induce apoptosis, while there was another group that received 30 ng/mL exogenous bFGF together with H_2_O_2_ as the DMEM‐HG+bFGF+H_2_O_2_ group. The cells were then washed twice with PBS, trypsinized and finally incubated with 500 μL of JC‐1 (5 mmol/L) for 30 minutes at 37°C. The cells were then centrifuged at 300 *g* for 5 minutes, and washed twice with 1× incubation buffer. The cells were resuspended in 500 μL of 1× incubation buffer. The Δψm was measured by detecting the red and green fluorescent emissions by flow cytometry, setting the excitation wavelength at 488 nm and the emission wavelength at 530 nm.

### 2.9 Measurement of H_2_O_2_‐induced reactive oxygen species

The levels of reactive oxygen species (ROS) in the H9C2 cardiomyocytes were measured using a 2′7′‐dichlorofluorescin diacetate (DCFH‐DA) assay kit. Approximately 1 × 10^6^ cells/well were seeded in 6‐well plates and treated with DMEM‐HG or iPS‐CM. The cells were incubated at 37°C in a 5% CO_2_ incubator for 24 hours. The cells were then treated with 200 μmol/L of H_2_O_2_ for 24 hours to induce apoptosis, while another group received 30 ng/mL exogenous bFGF together with H_2_O_2_ as the DMEM‐HG+bFGF+H_2_O_2_ group. The cells were then suspended in 200 μL DCFH‐DA for 20 minutes at 37°C in the dark. The cells were washed twice with PBS, and the fluorescence intensity was detected by flow cytometry.

### 2.10 Western blot analysis

H9C2 cardiomyocytes from different groups were washed with PBS and lysed for 30 minutes on ice in RIPA buffer (Bocai Biotechnology, Shanghai, China) containing a protease inhibitor cocktail (Amyjet Scientific Co., Ltd, Wuhan, China). The cells were then centrifuged at 12 000 *g* for 15 minutes at 4°C. Some cells were partitioned into nuclear and cytoplasmic fractions by using nuclear and cytoplasmic protein extraction kit (Beyotime Biotech Inc., Nantong, Jiangsu, China) according to the manufacturer's instructions. The protein concentration was measured according to the manufacturer's protocol using a BCA assay kit (Takara Bio Inc., Shiga, Japan). Then, 40 μg of protein was then subjected to 10% SDS‐PAGE and transferred to polyvinylidene fluoride membranes. The membranes were then blocked with 5% non‐fat milk in Tris‐buffered saline containing 0.05% Tween 20 (TBST) overnight at 4°C. Next, the membranes were incubated with primary antibodies (listed in Table 1) overnight at 4°C. The following day, the membranes were washed five times with TBST, and incubated with horseradish peroxidase‐conjugated secondary antibodies (1:5000; Bioword, MN, USA) for 2 hours at room temperature. The protein bands were detected by enhanced chemiluminescence (Pierce Chemical Co., IL, USA) and quantitated using ImageJ. The ratio of the expression of target proteins was determined after normalising to the β‐Actin level.

**Table 1:** Antibodies

Antibody	Species	Vendor (City, State, catalogue)	WB
β‐Actin	Rabbit	Cell Signaling Technology (Danvers, MA)	1:1000
BAX	Rabbit	Cell Signaling Technology	1:1000
BCL2	Rabbit	Cell Signaling Technology	1:1000
Histone 3	Rabbit	Cell Signaling Technology	1:1000
phospho‐β‐catenin (Ser675)	Rabbit	Cell Signaling Technology	1:1000
β‐catenin	Rabbit	Cell Signaling Technology	1:1000
Cyclin D1	Mouse	Santa Cruz (Santa Cruz, CA)	1:2000
c‐Myc	Mouse	Santa Cruz	1:2000
Survivin	Mouse	Santa Cruz	1:2000
LC3‐I/II	Rabbit	Cell Signaling Technology	1:1000
Beclin‐1	Rabbit	Cell Signaling Technology	1:1000
P62	Rabbit	Cell Signaling Technology	1:1000
P53	Mouse	Cell Signaling Technology	1:1000

### 2.11 Cell cycle assay

H9C2 cardiomyocytes (1 × 10^5^ cells/well) were cultured in 6‐well plates with DMEM‐HG, iPS‐CM and DMEM‐HG+bFGF (30 ng/mL). The cells were incubated for 24 hours at 37°C in a 5% CO_2_ incubator. The cells were then harvested and fixed in 75% ethanol at 4°C overnight. The following day, the cells were washed with PBS, and stained with PI in the dark for 30 minutes at room temperature. The stained cells were analysed using flow cytometry.

### 2.12 EdU staining

A Click‐iT^®^ EdU kit (Invitrogen, CA, USA) was used according to the manufacturer's instructions. H9C2 cardiomyocytes at a density of 1 × 10^5^ cells/well were seeded in 6‐well plates with DMEM‐HG, iPS‐CM and DMEM‐HG+bFGF (30 ng/mL), and were then incubated at 37°C in a 5% CO_2_ incubator for 24 hours. Then, 20 μmol/L EdU solutions were added to the cells, and incubated at 37°C for another 2 hours. The H9C2 cardiomyocytes were collected through digestion and centrifugation, and was fixed with 4% paraformaldehyde for 15 minutes and permeabilised using Triton X‐100 solution for 30 minutes at room temperature in the dark. The fixed cells were then stained with Click‐iT™ reaction mixture, and the cell nuclei were stained with 1 μg/mL DAPI in the dark, at room temperature for 30 minutes. The stained H9C2 cardiomyocytes were observed under an inverted fluorescence microscope and the cell fluorescence was quantified using flow cytometry.

### 2.13 SA‐β‐gal staining

H9C2 cardiomyocytes (1 × 10^6^ cells/well) were cultured in 6‐well plates with DMEM‐HG and iPS‐CM, and were then incubated at 37°C in a 5% CO_2_ incubator for 24 hours. To induce cell senescence, the cells were treated with 100 μmol/L H_2_O_2_ for 48 hours, while a group was treated with 30 ng/mL exogenous bFGF together with H_2_O_2_ as the DMEM‐HG+bFGF+H_2_O_2_ group. The medium was then removed and the cells were washed with PBS before being fixed for 15 minutes with β‐galactosidase fixative at room temperature. The cells were then washed with PBS three times and then stained with SA‐β‐gal stain solution overnight at 37°C. The cells were observed under an inverted fluorescence microscope. The SA‐β‐gal‐positive cells stained blue were expressed as a percentage of total cells. Quantification of the results was carried out by ImageJ software.

### 2.14 Measurement of MDA, SOD and GSH

H9C2 cardiomyocytes (1 × 10^6^ cells/well) were plated into 6‐well plates and cultured with DMEM‐HG and iPS‐CM at 37°C in a 5% CO_2_ incubator for 24 hours. Then, 200 μmol/L H_2_O_2_ was added to the respective wells, while there was a group that was treated with 30 ng/mL exogenous bFGF together with H_2_O_2_ as the DMEM‐HG+bFGF+H_2_O_2_ group. The cells were incubated at 37°C in a 5% CO_2_ incubator for another 24 hours. The cells were then collected and sonicated with phosphate buffer (pH 6.8) containing 1.0 mmol/L phenylmethylsulfonyl fluoride to obtain cell homogenates. The homogenates were centrifuged at 1200 *g* for 10 minutes at 4°C. The supernatants were collected and used for determining cellular MDA, SOD and GSH levels using commercially available assay kits (Jiancheng Biochemical Inc.). The MDA level was determined by evaluating the thiobarbituric acid reacting substances at a wavelength of 532 nm using an Infinite M200 microplate reader (Tecan Group Ltd., Mannedorf, Switzerland). The SOD activity was examined using the xanthine oxidase method, with absorbance set at 450 nm. The GSH levels were measured based on the Ellman method.^30^ The cell homogenate was mixed with reaction buffer (pH 8.0) and 5, 5′‐dithiobis‐(2‐nitrobenzoic acid) for 5 minutes. The colour change was measured at a wavelength of 412 nm. All values were normalised according to the total protein concentration of the respective samples.

### 2.15 Enzyme‐linked immunosorbent assay

An enzyme‐linked immunosorbent assay (ELISA) kit was used to determine the levels of bFGF in DMEM‐HG, iPS‐CM and mTeSR1. Briefly, 200 μL samples and 50 μL assay diluent were added to pre‐coated wells of 96‐well plates, and incubated at room temperature for 2 hours. The plates were then washed five times with washing buffer. Each well was treated with 100 μL of peroxidase‐conjugated IgG anti‐bFGF solution for 2 hours at room temperature. The plates were washed again five times with washing buffer. Then, 100 μL of substrate buffers were added into each well and incubated for 30 minutes at room temperature in the dark. Finally, the enzyme reaction was quenched with 50 μL of stop solution. The assays were analysed using a microplate reader at a wavelength of 550 nm with a correction wavelength of 450 nm.

### 2.16 Statistical analysis

The collected data were statistically analysed using GraphPad Prism software (version 6; GraphPad Software Inc., San Diego, CA, USA). Comparisons of different groups were performed using one‐way ANOVA followed by Tukey's test. The values were expressed as the mean ± SD, and *P* < 0.05 was considered to be statistically significant.

## 3 RESULTS

### 3.1 The optimal concentration of iPS‐CM and H_2_O_2_ for H9C2 cardiomyocyte proliferation and apoptosis respectively

**Figure 1:**
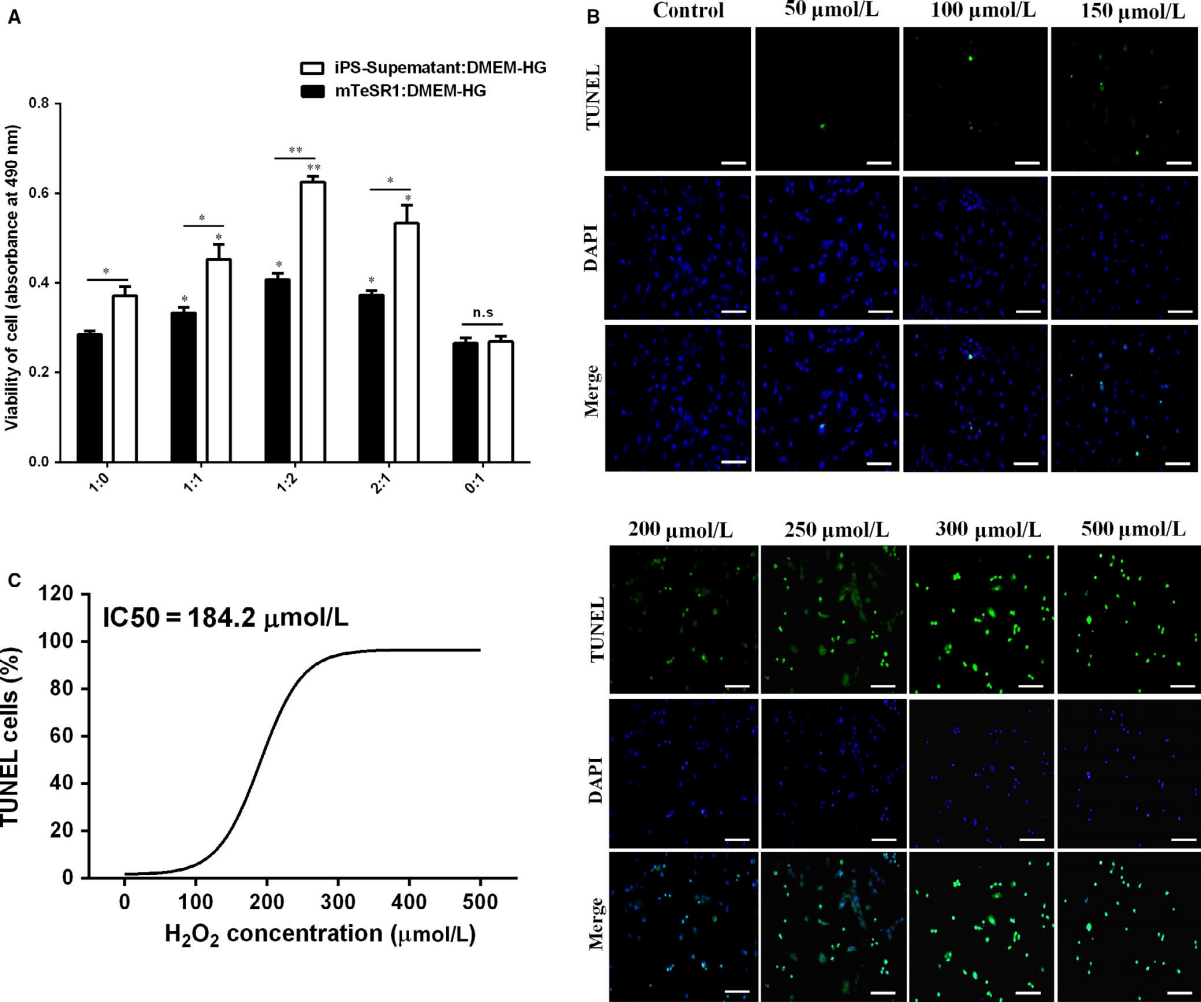
Exploration the optimal proportion of DMEM‐HG:iPS‐supernatant and apoptosis model of H_2_O_2_‐induced H9C2 cardiomyocytes. A, The proliferation viabilities of H9C2 cardiomyocytes in different proportion of DMEM‐HG:iPS‐supernatant or mTeSR1:DMEM‐HG were analysed by CCK‐8. B, The TUNEL staining of H9C2 cardiomyocytes exposed to H_2_O_2_ at different concentrations under an inverted fluorescence microscope. C, The quantity of positive cells (green fluorescence) using Image‐Pro Plus 6.0 software. Mean ± SE, n = 5. *P < 0.05, **P < 0.01 designate significant differences when compared to control (1:0 or 0:1). The n.s designates no significant difference. Scale bars 100 μm

A CCK‐8 kit was used to determine the optimal concentration of iPS supernatant:DMEM‐HG as the iPS‐CM. H9C2 cardiomyocytes were treated with different ratios of iPS supernatant:DMEM‐HG or mTeSR1:DMEM‐HG (1:0, 1:1, 1:2, 2:1 and 0:1) for 24 hours. The results showed that the absorbance was the most prominent with iPS supernatant:DMEM‐HG or mTeSR1:DMEM‐HG ratio of 1:2, and the absorbance of iPS supernatant:DMEM‐HG at 1:2 was significantly greater than that of mTeSR1:DMEM‐HG, indicating that cell viability was optimal at a 1:2 ratio of iPS supernatant:DMEM‐HG as the iPS‐CM (Figure 1A). To establish the optimal apoptosis model, H9C2 cardiomyocytes were exposed to different concentrations of H_2_O_2_ (0, 50, 100, 150, 200, 250, 300 and 350 μmol/L). The TUNEL staining showed that H9C2 cardiomyocytes subjected to H_2_O_2_ (≤150 μmol/L) had little apoptotic cells (green fluorescence), but H9C2 cardiomyocytes subjected to H_2_O_2_ (≥200 μmol/L) showed a large number of positive staining cells (Figure 1B). The quantitative results showed that the IC50 (semi‐apoptotic concentration) was 184.2 μmol/L which was near to 200 μmol/L, so 200 μmol/L H_2_O_2_ was selected as the optimal concentration for the following apoptosis model (Figure 1C).

### 3.2 iPS‐CM inhibited H_2_O_2_‐induced apoptosis of H9C2 cardiomyocytes

**Figure 2:**
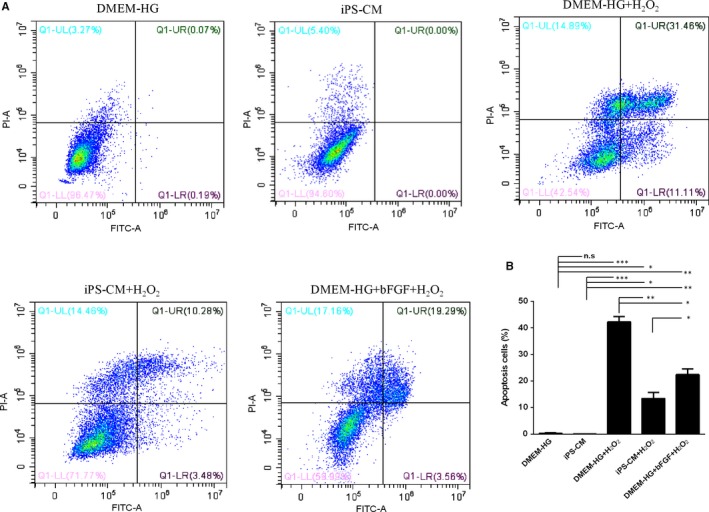
iPS‐CM inhibited the apoptosis of H_2_O_2_‐induced H9C2 cardiomyocytes. A, Annexin V and propidium iodide (PI) assay was used to analyse the cell apoptosis in different groups. B, Quantification of the Annexin V and PI assay. Mean ± SE, n = 3. *P < 0.05, **P < 0.01, ***P < 0.001, designate significant differences. The n.s designates no significant difference

Annexin V and PI assays were used to evaluate apoptosis in different groups, namely, DMEM‐HG, iPS‐CM, DMEM‐HG+H_2_O_2_, iPS‐CM+H_2_O_2_ and DMEM‐HG+bFGF+H_2_O_2_. The percentage of apoptotic cells was calculated from the Q1‐LR (early stage of apoptosis) and Q1‐UR (late stage of apoptosis). The results showed that iPS‐CM decreased the apoptosis ratio of H_2_O_2_‐treated H9C2 cardiomyocytes, and bFGF added to DMEM‐HG also rescued H_2_O_2_‐treated H9C2 cardiomyocytes (Figure 2A). The DMEM‐HG and iPS‐CM groups almost had nearly no apoptotic cells, with only 0.26 ± 0.07% and 0.00 ± 0.00% apoptotic cells respectively. The DMEM‐HG+H_2_O_2_ group had 42.57 ± 3.44% apoptotic cells, which was considerably higher than that in the DMEM‐HG group. The apoptotic ratio in the iPS‐CM+H_2_O_2_ group was 13.76 ± 0.86%, which was greater than that of the iPS‐CM group but lower than that of the DMEM‐HG+H_2_O_2_ group. Furthermore, the apoptotic ratio in the DMEM‐HG+bFGF+H_2_O_2_ group was 22.75 ± 1.73%, which was greater than that of the DMEM‐HG, iPS‐CM and iPS‐CM+H_2_O_2_ groups but less than that of the DMEM‐HG+H_2_O_2_ group (Figure 2B). This result demonstrated that iPS‐CM inhibited H_2_O_2_‐induced apoptosis of H9C2 cardiomyocytes and that bFGF also partially rescued H_2_O_2_‐treated H9C2 cardiomyocytes.

**Figure 3:**
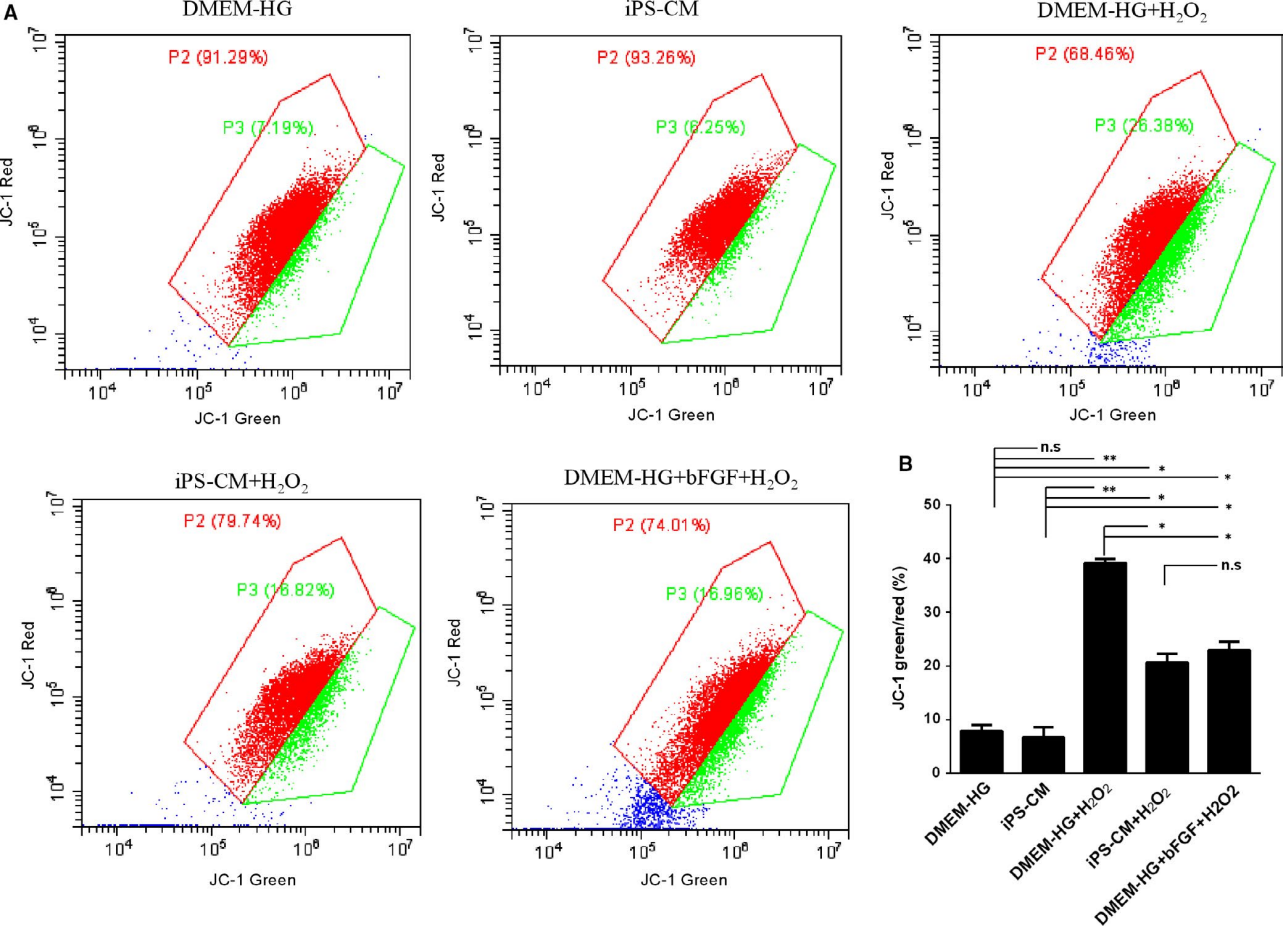
iPS‐CM inhibited the loss of mitochondrial membrane potential (△Ψm) in H_2_O_2_‐induced H9C2 cardiomyocytes. A, Mitochondrial membrane potential (△Ψm) assay was used to analyse the loss of △Ψm in different groups. B, Quantification of △Ψm. Mean ± SE, n = 3. *P < 0.05, **P < 0.01 designate significant differences. The n.s designates no significant difference

As the loss of △Ψm is associated with early apoptosis, JC‐1 assay was used to analyse the effects of iPS‐CM on the loss of △Ψm in H_2_O_2_‐treated H9C2 cardiomyocytes. The △Ψm of apoptotic cells was less than that of living cells. The ratio of JC‐1‐green/red indirectly reflects apoptosis. A low ratio indicates less apoptosis than a high ratio. The loss of △Ψm in H9C2 cardiomyocytes with H_2_O_2_ treatment increased noticeably compared with that of DMEM‐HG and iPS‐CM groups, and was inhibited by iPS‐CM or bFGF treatments (Figure 3A). The data showed that the ratios of JC‐1‐green/red in the DMEM‐HG and iPS‐CM groups were 7.87 ± 0.45% and 6.70 ± 0.38%, respectively, which were less than those of the DMEM‐HG+H_2_O_2_ (38.53 ± 4.17%), iPS‐CM+H_2_O_2_ (21.09 ± 2.53%) and DMEM‐HG+bFGF+H_2_O_2_ (22.92 ± 3.08%) groups. The ratio of JC‐1‐green/red in the iPS‐CM+H_2_O_2_ group was also less than that of the DMEM‐HG+H_2_O_2_ group and was similar to that of the DMEM‐HG+bFGF+H_2_O_2_ group, which was also less than that of the DMEM‐HG+H_2_O_2_ group (Figure 3B). This result showed that iPS‐CM inhibited the loss of △Ψm in H_2_O_2_‐treated H9C2 cardiomyocytes, and bFGF also reduced the loss of △Ψm in H_2_O_2_‐treated H9C2 cardiomyocytes.

**Figure 4:**
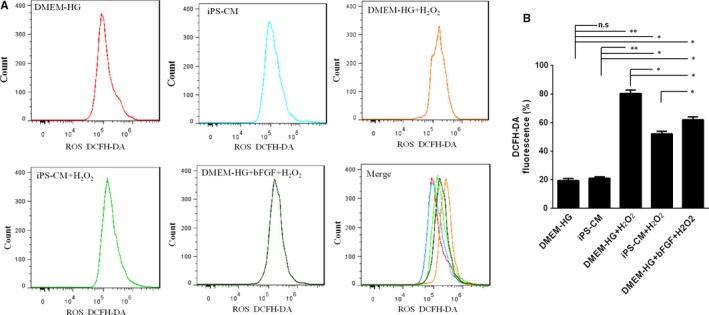
iPS‐CM inhibited the generation of intracellular reactive oxygen species (ROS) in H_2_O_2_‐induced H9C2 cardiomyocytes. A, Induced reactive oxygen species (ROS) assay was used to analyse the generation of ROS in different groups. B, Quantification of intracellular ROS. Mean ± SE, n = 3. *P < 0.05, **P < 0.01 designate significant differences. The n.s designates no significant difference

DCFH‐DA was used to evaluate the effects of iPS‐CM on the generation of ROS in H_2_O_2_‐treated H9C2 cardiomyocytes. The value of DCFH‐DA fluorescence reflects the generation of ROS. The generation of ROS in H9C2 cardiomyocytes after H_2_O_2_ administration substantially increased compared with that of the DMEM‐HG and iPS‐CM groups, and iPS‐CM and bFGF decreased H_2_O_2_‐induced ROS generation (Figure 4A). The results showed that the values of DCFH‐DA fluorescence in the DMEM‐HG and iPS‐CM groups were 21.03 ± 1.79% and 21.59 ± 0.87%, respectively, which were less than those of the DMEM‐HG+H_2_O_2_ (78.21 ± 5.77%), iPS‐CM+H_2_O_2_ (54.36 ± 3.64%) and DMEM‐HG+bFGF+H_2_O_2_ (60.32 ± 4.17%) groups. Moreover, the value of DCFH‐DA fluorescence in the iPS‐CM+H_2_O_2_ group was less than that in the DMEM‐HG+H_2_O_2_ group. Furthermore, the value of DCFH‐DA fluorescence in the DMEM‐HG+bFGF+H_2_O_2_ group was less than that in the DMEM‐HG+H_2_O_2_ group but was greater than that in the iPS‐CM+H_2_O_2_ group (Figure 4B). This result demonstrated that iPS‐CM inhibited the generation of ROS in H_2_O_2_‐treated H9C2 cardiomyocytes and that bFGF also partially decreased the generation of ROS in H_2_O_2_‐treated H9C2 cardiomyocytes.

### 3.3 The effects of iPS‐CM on BAX and BCL‐2 expression in H_2_O_2_‐treated H9C2 cardiomyocytes

**Figure 5:**
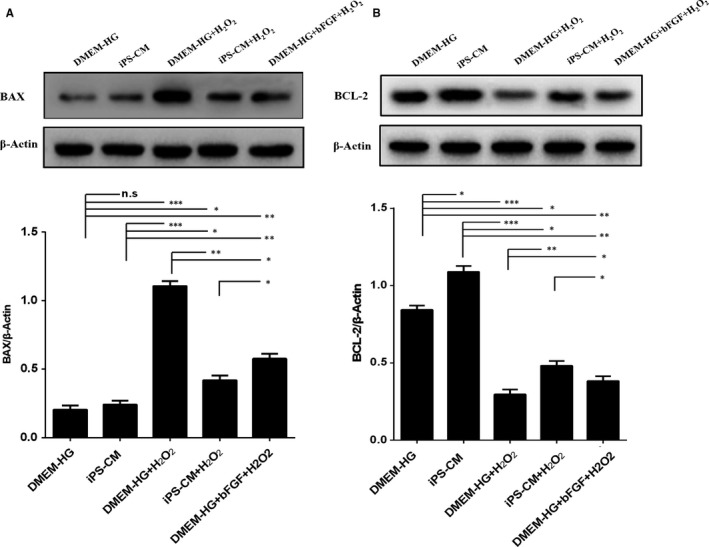
iPS‐CM suppressed BAX upregulation and BCl‐2 downregulation of H_2_O_2_‐induced H9C2 cardiomyocytes. A, The protein expression of BAX in different groups. B, The protein expression of BCL‐2 in different groups. Mean ± SE, n = 3. *P < 0.05, **P < 0.01, ***P < 0.001, designate significant differences. The n.s designates no significant difference

To investigate the potential molecular mechanisms involved in the anti‐apoptosis activity caused by iPS‐CM on H_2_O_2_‐treated H9C2 cardiomyocytes, the expression levels of apoptotic signalling molecules in the mitochondria, including the apoptotic protein BAX and the anti‐apoptotic protein BCL‐2, were analysed by Western blot in different groups. When H9C2 cardiomyocytes were treated with H_2_O_2_, BAX expression was up‐regulated in the DMEM‐HG+H_2_O_2_ and iPS‐CM+H_2_O_2_ groups, and the expression level in the iPS‐CM+H_2_O_2_ group was less than that in the DMEM‐HG+H_2_O_2_ group. The expression level of BAX in the DMEM‐HG+bFGF+H_2_O_2_ group was less than that in the DMEM‐HG+H_2_O_2_ group but was greater than that in the DMEM‐HG, iPS‐CM and iPS‐CM+H_2_O_2_ groups (Figure 5A). The trend of BCL‐2 expression was almost opposite to that of BAX expression. Compared with the DMEM‐HG and iPS‐CM groups, H_2_O_2_ significantly inhibited the expression of BCL‐2 in the DMEM‐HG+H_2_O_2_ and iPS‐CM+H_2_O_2_ groups. In addition, the expression level of BCL‐2 in the iPS‐CM+H_2_O_2_ group was greater than that in the DMEM‐HG+H_2_O_2_ and DMEM‐HG+bFGF+H_2_O_2_ groups, and the expression level in the DMEM‐HG+bFGF+H_2_O_2_ group was also greater than that in the DMEM‐HG+H_2_O_2_ group (Figure 5B). These results indicated that iPS‐CM or bFGF protected H9C2 cardiomyocytes from apoptosis by suppressing the expression of BAX while promoting the expression of BCL‐2.

### 3.4 iPS‐CM promoted the proliferation of H9C2 cardiomyocytes

**Figure 6:**
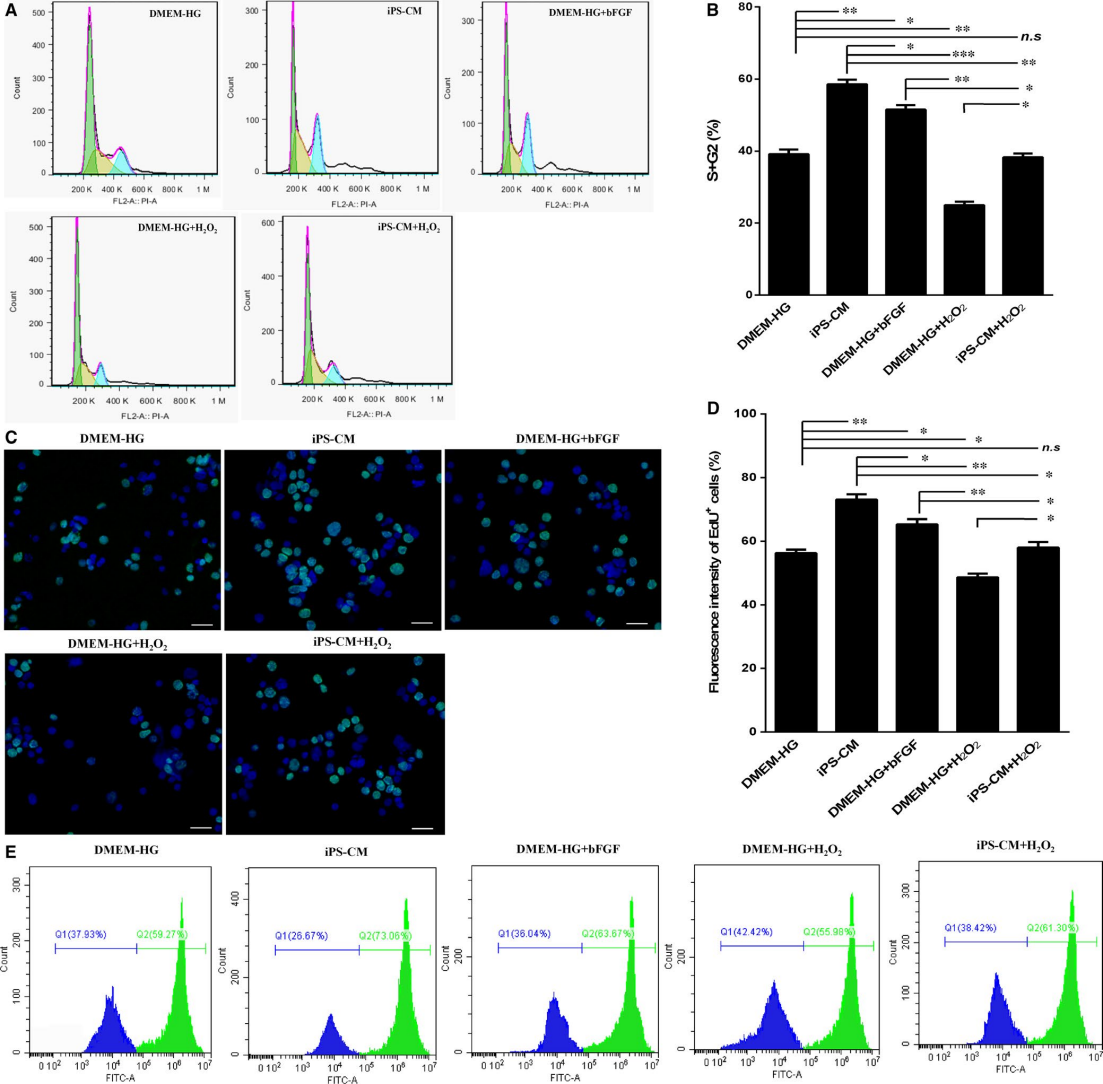
iPS‐CM promotes the proliferation viability of H9C2 cardiomyocytes. A, The cell cycle assay was conducted to analyse the proliferation viability in different groups. B, Quantification of the cell cycle distribution. C, EdU staining images at an inverted fluorescence microscope. D, Quantification of the EdU positive cells. E, Fluorescent density of EdU positive cells were detected by flow cytometry assays. Mean ± SE, n = 3. *P < 0.05, **P < 0.01, ***P < 0.001 designate significant differences. The n.s designates no significant difference. Scale bars 100 μm

A cell cycle assay was used to determine the cell proliferation in the different groups. Cell proliferation occurs in the S and G2 phases. iPS‐CM and bFGF promoted the proliferation of H9C2 cardiomyocytes. Moreover, iPS‐CM could also promote H9C2 cardiomyocyte proliferation under H_2_O_2_ treatment (Figure 6A). The quantification data showed that the percentage of cells entering the S and G2 phases in the DMEM‐HG group was 40.55 ± 1.16%, which was less than that in the iPS‐CM (57.72 ± 2.76%) and DMEM‐HG+bFGF (50.56 ± 2.04%) groups, and the percentage of proliferating cells in the DMEM‐HG+bFGF group was also less than that in the iPS‐CM group. In addition, the percentage of cells entering the S and G2 phases in the DMEM‐HG+H_2_O_2_ group was 26.12 ± 0.83%, which was less than that in the iPS‐CM+H_2_O_2_ (37.43 ± 1.05%) group (Figure 6B). EdU can be incorporated into cellular DNA during DNA replication, and it is widely used to analyse cell proliferation. Under an inverted fluorescence microscope, the proliferating cells appeared fluorescent green (Figure 6C). The results of EdU staining showed that the number of positive cells with green fluorescence in the DMEM‐HG group was less than that in the iPS‐CM and DMEM‐HG+bFGF groups, and the number of cells in the DMEM‐HG+bFGF group was also less than that in the iPS‐CM group. Meanwhile, the number of green fluorescence cells in the DMEM‐HG+H_2_O_2_ group was less than that in the iPS‐CM+H_2_O_2_ group (Figure 6D). Furthermore, the flow cytometry assays also demonstrated that the green fluorescent cell densities in the DMEM‐HG group were less than those in the iPS‐CM and DMEM‐HG+bFGF groups, and the green fluorescent densities in the DMEM‐HG+bFGF group were also less than those in the iPS‐CM group as well as the green fluorescent densities in the DMEM‐HG+H_2_O_2_ group were also less than those in the iPS‐CM+H_2_O_2_ group (Figure 6E). These results suggested that iPS‐CM could promote the proliferation of H9C2 cardiomyocytes even under the H_2_O_2_ administration.

### 3.5 iPS‐CM inhibited H_2_O_2_‐induced cell senescence and oxidative stress in H9C2 cardiomyocytes

**Figure 7:**
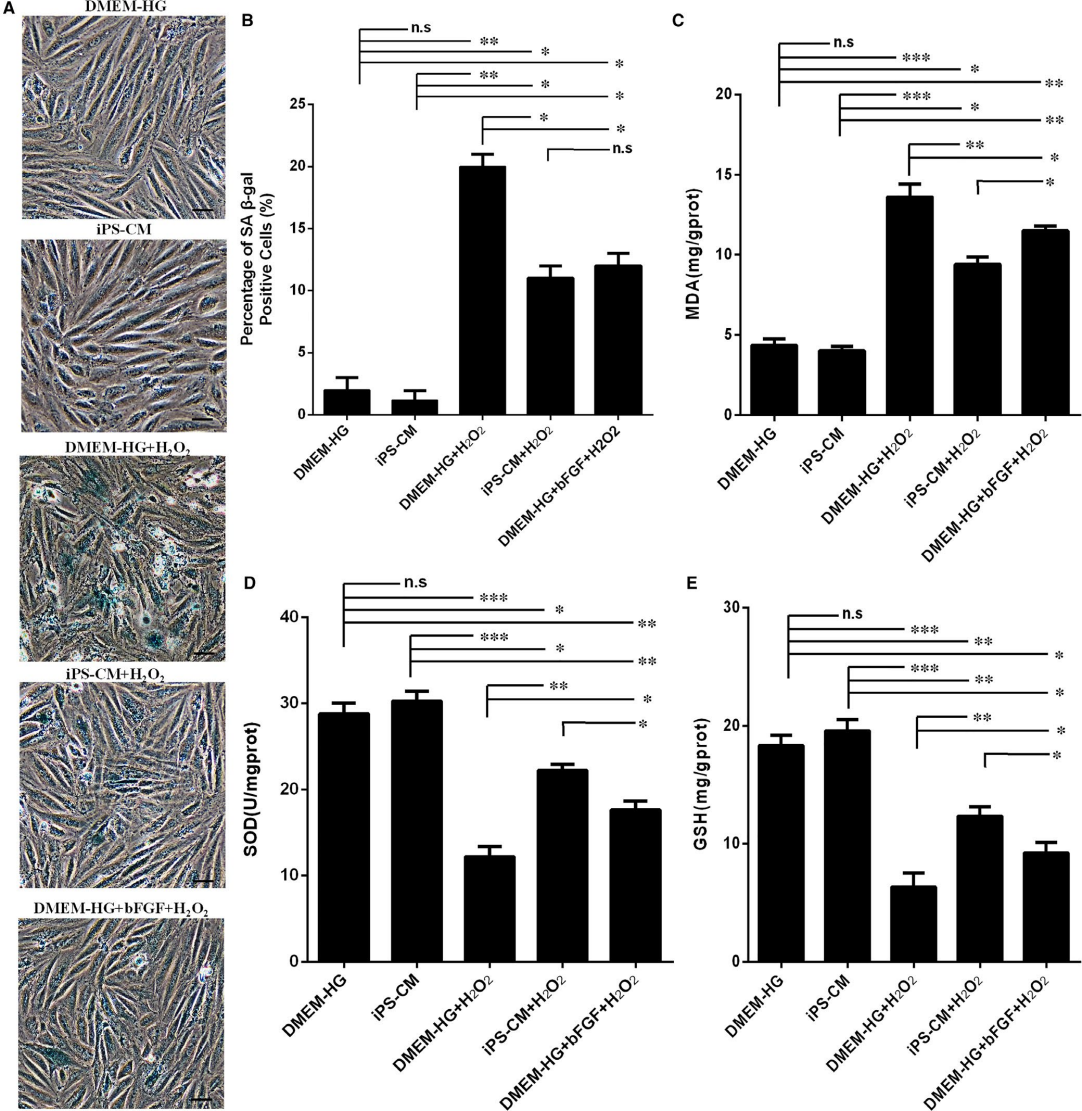
iPS‐CM alleviated H_2_O_2_‐induced SA‐β‐gal activity and oxidative stress of H9C2 cardiomyocytes. A, The images of SA‐β‐gal staining of the H9C2 cardiomyocytes exposed to 100 μmol/L H_2_O_2_ with different treatments under an inverted microscope. B, Quantification of the SA‐β‐gal positive cells with ImageJ software. C, The MDA levels of the H9C2 cardiomyocytes exposed to 200 μmol/L H_2_O_2_ with different treatments. D, The SOD levels of the H9C2 cardiomyocytes exposed to 200 μmol/L H_2_O_2_ with different treatments. E, The GSH levels of the H9C2 cardiomyocytes exposed to 200 μmol/L H_2_O_2_ with different treatments. Mean ± SE, n = 3. *P < 0.05, **P < 0.01, ***P < 0.001, designate significant differences. The n.s designates no significant difference. Scale bars 100 μm

Cell senescence is defined as the irreversible cell cycle arrest of mitotic cells, leading to a change in the cellular phenotype. Cell senescence can be induced by external stimuli, such as oxidative stress. H9C2 cardiomyocytes were exposed to a low dose of H_2_O_2_ (100 μmol/L) for 48 hours. SA‐β‐gal staining was performed to investigate H_2_O_2_‐induced cell senescence, and blue staining indicated senescent cells. Some blue stained cells appeared more flattened (Figure 7A). This observation is in accordance with previous studies.^31^ The quantification results showed that the low dose of H_2_O_2_‐induced H9C2 cardiomyocyte senescence, but treatment with iPS‐CM or bFGF suppressed cell senescence. The percentages of SA‐β‐gal positive cells in the DMEM‐HG and iPS‐CM groups were very low, and both percentages were less than those in the DMEM‐HG+H_2_O_2_, iPS‐CM+H_2_O_2_ and DMEM‐HG+bFGF+H_2_O_2_ groups. Moreover, the percentage of SA‐β‐gal‐positive cells in the iPS‐CM+H_2_O_2_ group was also less than that in the DMEM‐HG+H_2_O_2_ group and was similar to that in the DMEM‐HG+bFGF+H_2_O_2_ group (Figure 7B). Therefore, we deduced that iPS‐CM or bFGF suppressed H_2_O_2_‐induced cell senescence in H9C2 cardiomyocytes.

In addition, H_2_O_2_‐induced oxidative stress can be inhibited by antioxidants. SOD and GSH are natural antioxidants found in the cells. These antioxidants likely protect cells from oxidative stress. However, MDA is the end‐product of lipid peroxidation and therefore can be found at high levels in the state of oxidative stress. As shown in Figure 7C, compared with the DMEM‐HG and iPS‐CM groups, H_2_O_2_ treatment led to a significant increase in MDA levels. However, iPS‐CM and bFGF significantly decreased the H_2_O_2_‐induced MDA levels. Furthermore, the levels of the antioxidant SOD and GSH were high in the DMEM‐HG and iPS‐CM groups. However, these levels considerably decreased when H9C2 cardiomyocytes were treated with H_2_O_2_ alone, and iPS‐CM or bFGF treatment could remit the loss of antioxidant SOD and GSH (Figure 7D,E). These results demonstrated that iPS‐CM or bFGF inhibited H_2_O_2_‐induced oxidative stress in H9C2 cardiomyocytes.

### 3.6 The potential mechanism underlying the promotion of H9C2 cardiomyocyte anti‐apoptosis and proliferation by iPS‐CM

**Figure 8:**
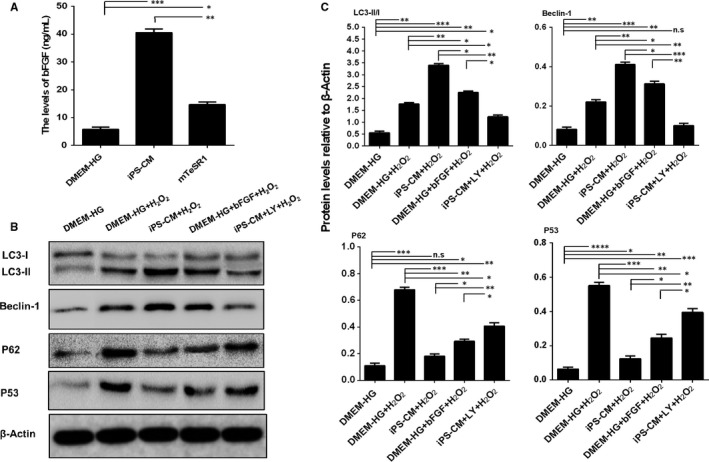
The potential mechanisms underlying the promotion of H9C2 cardiomyocyte anti‐apoptosis by iPS‐CM. A, Quantification of the bFGF levels of medium in different groups using ELISA. B, Assessment of protein expression levels of LC3 I/II, Beclin‐1, P62 and P53 in different groups using Western blot assays. C, Quantification of Western blot assays. Mean ± SE, n = 3. *P < 0.05, **P < 0.01, ***P < 0.001, ****P < 0.0001, designate significant differences. The n.s designates no significant difference

To elucidate the underlying mechanism of the anti‐apoptotic and proliferative properties of iPS‐CM on H9C2 cardiomyocytes, we first analysed the levels of different growth factors, such as bFGF, IGF‐1 and VEGF in DMEM‐HG, iPS‐CM and mTeSR1 using ELISA. Only bFGF, but not IGF‐1 and VEGF (data not shown), showed significant differences among these groups, and the level of bFGF in iPS‐CM was significantly greater than that in DMEM‐HG or mTeSR1 (Figure 8A). Based on this result, exogenous bFGF (30 ng/mL) was added to the DMEM‐HG to establish a new group that mimicked the iPS‐CM group, and the underlying signalling pathways were further investigated.

To examine the role of iPS‐CM in the activation of autophagy flux to suppress apoptosis of H_2_O_2_‐treated cardiomyocytes, the levels of autophagy related proteins, such as LC3‐I/II, Beclin‐1 and P62, and the apoptotic protein P53 were assessed in the different groups by Western blot (Figure 8B). P62, a substrate of the autophagic process, is a marker of autophagic flux, and the accumulation of P62 indicates disrupted autophagy flux.^32^ The ratio of LC3 II/I and the level of Beclin‐1 were significantly up‐regulated but the expression of P62 and P53 was inhibited by iPS‐CM treatment in H_2_O_2_‐treated H9C2 cardiomyocytes compared with the DMEM‐HG+H_2_O_2_ group. Additionally, the mimic group (DMEM‐HG+bFGF) achieved the same effects as iPS‐CM in H_2_O_2_‐treated H9C2 cardiomyocytes. However, the up‐regulated autophagic proteins LC3 and Beclin‐1, and the down‐regulated autophagic flux protein P62 and the apoptotic protein P53 were also inhibited by LY294002 (25 μmol/L, Sigma), a known inhibitor of autophagy flux.^33^ In addition, all these proteins were slightly expressed in the H9C2 cardiomyocytes without any treatment (Figure 8C). These results suggested that iPS‐CM partially depended on endogenous bFGF to suppress the apoptosis of H9C2 cardiomyocytes by activating the autophagy flux pathway.

**Figure 9:**
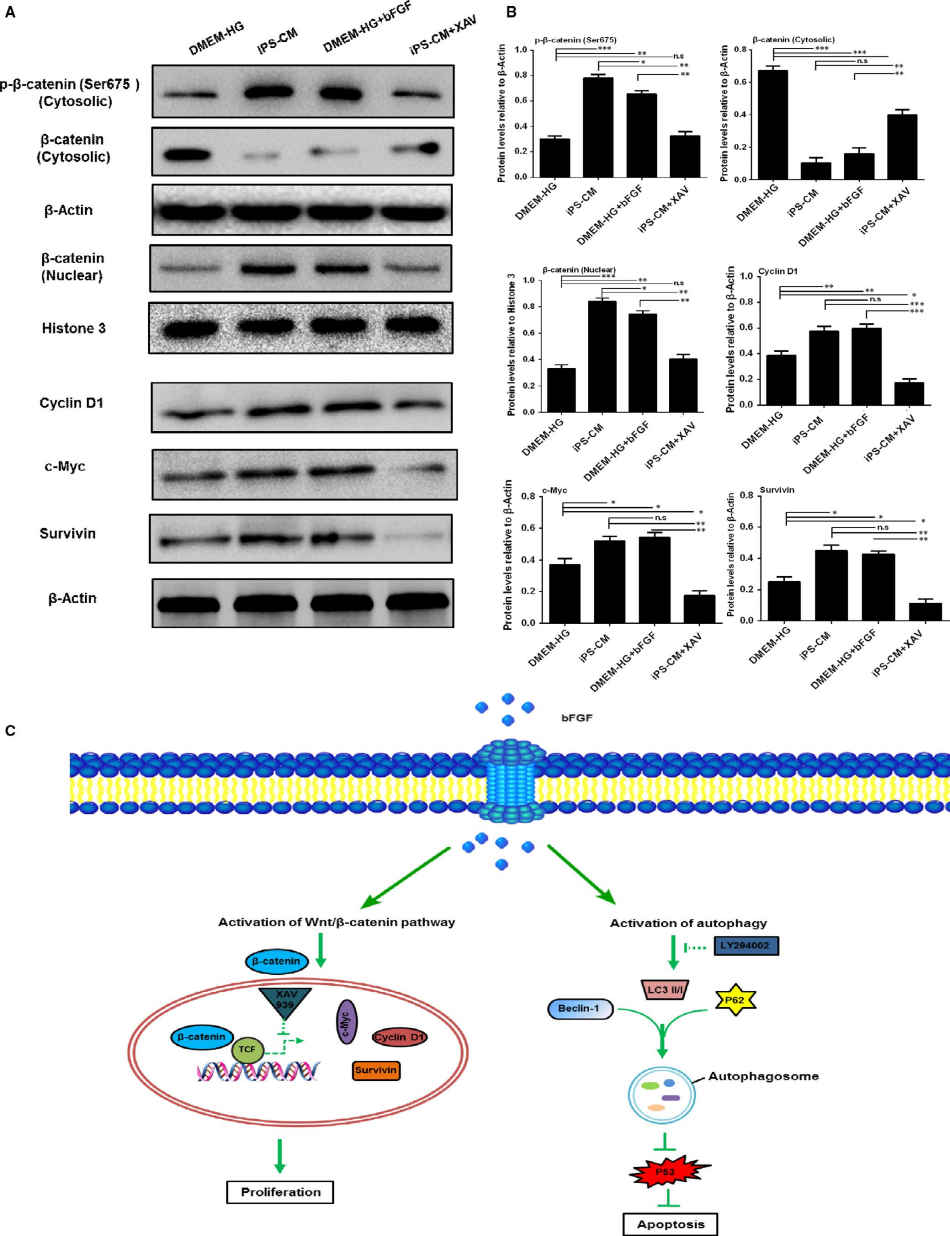
The potential mechanisms underlying the promotion of H9C2 cardiomyocyte proliferation by iPS‐CM. A, Assessment of protein expression levels of p‐β‐catenin (ser 675), β‐catenin (nuclear protein), β‐catenin (cytosolic protein), Cyclin D1, c‐Myc and Survivin in different groups using Western blot assays. B, Quantification of Western blot assays. C, Diagram of the potential mechanisms underlying the promotion of H9C2 cardiomyocytes anti‐apoptosis and proliferation by iPS‐CM. Mean ± SE, n = 3. *P < 0.05, **P < 0.01, ***P < 0.001, designate significant differences. The n.s designates no significant difference

A classical signalling pathway for cell proliferation is the Wnt/β‐catenin pathway.^34^ Subsequently, we investigated the underlying mechanism for the proliferation effect of iPS‐CM. The Western blot results showed a greater level of p‐β‐catenin (ser 675), β‐catenin (nuclear protein) other than β‐catenin (cytosolic protein), and its target proteins Cyclin D1, c‐Myc and Survivin in the iPS‐CM group than that in the DMEM‐HG group (Figure 9A). A similar set of results was obtained in the mimic group (DMEM‐HG+bFGF). When XAV939 (10 μmol/L, Sigma), a known inhibitor of the Wnt/β‐catenin pathway, was added to the iPS‐CM, the expression of those proteins was down‐regulated (Figure 9B). The β‐catenin is a key downstream effector in the Wnt signalling pathway. Phosphorylation at ser675 induces β‐catenin accumulation in the nucleus and increases its transcriptional activity.^35^ Based on these results, we might deduce that iPS‐CM partially relied on endogenous bFGF to promote the proliferation of H9C2 cardiomyocytes through activation of the Wnt/β‐catenin signalling pathway. A schematic summary of the mechanisms underlying the promotion of the anti‐apoptotic and proliferative characteristics of H9C2 cardiomyocytes induced by iPS‐CM is illustrated in Figure 9C.

## 4 DISCUSSION

Myocardial infarction is a leading cause of death among all cardiovascular diseases.^3^ Approximately 20 million people die from cardiovascular diseases every year, and America alone has 1.5 million people suffering from myocardial infarction.^36^ In recent years, stem cell therapy for myocardial infarction has been the focus of numerous studies. Different cell types, such as mesenchymal stem cells (MSCs), cardiac progenitor cells and cardiosphere‐derived cells have been investigated.^37^, ^38^, ^39^, ^40^, ^41^, ^42^, ^43^ However, stem cell transplantation still has variable disadvantages, and the basic mechanisms that underlie effective cell therapy remain unclear.^44^ Based on our results, iPS‐CM was demonstrated to be a potential candidate for a novel therapy of myocardial infarction. The anti‐apoptotic ability of iPS‐CM may limit the extent of dead tissue, while its proliferative ability may encourage cardiac cells to proliferate and thus reduce the size of the infarct. By limiting oxidative stress, iPS‐CM prevents cells from entering the senescent phase, thus preventing the progression of an infarct.

Previous studies have shown that the proliferative ability of iPS‐CM is attributed to the products secreted by iPSCs in culture medium.^29^ Guo et al showed that the levels of bFGF and Activin A in iPS‐CM were greater than in fresh mTeSR1 medium.^29^ Villageois et al found that Activin A, which was secreted by hASCs isolated from various fat deposits of donors with different ages, promoted human multipotent adipose‐derived stem cell proliferation and adipocyte differentiation.^45^ In our study, bFGF levels in iPS‐CM were also significantly greater than those in DMEM‐HG and mTeSR1, which were determined using an ELISA assay. Based on this result, we added exogenous bFGF to DMEM‐HG to mimic the function of iPS‐CM.

Apoptosis is defined as highly regulated, programmed cell death that requires much energy and plays a key role in a variety of biological systems. Saraste et al showed that in myocardial samples obtained from acute myocardial infarction patients, a subset of cardiomyocytes underwent apoptosis.^46^ Apoptosis was more prominent in the border zones of recent infarctions.^47^, ^48^, ^49^ H_2_O_2_ is an ideal inductor for establishing an apoptosis model. In the present study, H9C2 cardiomyocytes were exposed to different concentrations of H_2_O_2_, and 200 μmol/L was determined for the apoptosis model. The mechanisms for apoptosis include direct damage to the mitochondria by ROS and indirect mitochondrial depolarisation by apoptosis‐related BCL‐2 family proteins. BAX is a pro‐apoptotic and pore‐forming cytoplasmic protein in the BCL‐2 family. Conversely, BCL‐2 is an anti‐apoptotic protein in the BCL‐2 family. In this study, H_2_O_2_‐treated H9C2 cardiomyocytes with iPS‐CM treatment underwent a lesser extent of apoptosis and the loss of Δψm, which is associated with apoptosis. Western blot further showed the up‐regulation of BCL‐2 and down‐regulation of BAX in cells in the iPS‐CM+H_2_O_2_ group. H_2_O_2_‐induced apoptosis typically results in the production of ROS in cells. After treatment with H_2_O_2_, H9C2 cardiomyocytes treated with iPS‐CM exhibited lower ROS levels than those in the DMEM‐HG group. In addition, exogenous bFGF added to DMEM‐HG exhibited anti‐apoptotic effects on H_2_O_2_‐treated H9C2 cardiomyocytes, and the level of bFGF in iPS‐CM was significantly greater than that in DMEM‐HG and mTeSR1. These results indicated that iPS‐CM partially depended on endogenous bFGF to inhibit the apoptosis of H_2_O_2_‐treated H9C2 cardiomyocytes.

Lian et al reported that iPS‐CM not only suppressed apoptosis, but also promoted cell proliferation by attenuating G1 phase arrest of the cell cycle in human adipose‐derived stem cells.^24^ In our study, the percentage of cells entering the S and G2 phases in H9C2 cardiomyocytes treated with iPS‐CM was greater than those cultured in DMEM‐HG, and exogenous bFGF added to DMEM‐HG also promoted the proliferation of H9C2 cardiomyocytes but was less effective than iPS‐CM. In addition, the number of EdU‐positive H9C2 cardiomyocytes, having iPS‐CM or exogenous bFGF treatments, was greater than that in the DMEM‐HG group. Therefore, we deduced that, along with inhibiting cellular apoptosis, iPS‐CM also partially depended on endogenous bFGF to promote the proliferation of H9C2 cardiomyocytes.

It has been previously demonstrated that the accumulation of oxidative stress contributes to the cell senescence.^50^, ^51^ Therefore, antioxidants play an important role in slowing biological ageing. Cell senescence is the irreversible growth arrest of individual mitotic cells. Senescent cells secrete pro‐inflammatory cytokines that affect neighbouring cells as well as the extracellular matrix. It has been speculated that only a small fraction of these senescent cells is required for significant tissue impairment or disease progression/development.^52^, ^53^, ^54^, ^55^ In this study, H9C2 cardiomyocytes exposed to low doses of H_2_O_2_ exhibited senescence characteristics, such as altered cell morphology, positive SA‐β‐gal staining and cell cycle arrest. However, iPS‐CM treatment significantly reduced cell senescence under chronic oxidative stress of H9C2 cardiomyocytes, and exogenous bFGF added to DMEM‐HG also inhibited the senescence of H_2_O_2_‐treated H9C2 cardiomyocytes but was less effective than iPS‐CM.

Cardiomyocyte apoptosis in myocardial infarction is mainly attributed to oxidative stress, the accumulation of calcium and mitochondrial dysfunction, among which oxidative stress plays an important role in cellular apoptosis.^56^ Oxidative stress is considered a key apoptotic stimulus in many cardiovascular diseases, which triggers myocyte apoptosis by up‐regulating pro‐apoptotic genes that are inhibited by antioxidants.^57^ Oxidative stress results from an imbalance between ROS production and elimination. Under normal conditions, H9C2 cardiomyocytes contain high levels of the antioxidants SOD and GSH, which likely play crucial roles in protecting the cells against oxidative stress. However, when cells are exposed to H_2_O_2_, these antioxidant defence systems are destroyed, and excessive ROS might accumulate, which could exceed the self‐scavenging ability of cells, resulting in oxidative stress that causes DNA and mitochondrial damage, protein modification and degradation and ultimately cell death.^58^ Furthermore, MDA is an end‐product of lipid peroxidation and is typically found in minimal levels in living cells. In our study, the results showed that H_2_O_2_ drastically accelerated lipid peroxidation while decreasing the levels of SOD and GSH antioxidants. Treatment with iPS‐CM or bFGF significantly attenuated the high MDA levels and enhanced SOD and GSH activity. These results suggested that iPS‐CM may partially depend on endogenous bFGF to inhibit oxidative stress in H_2_O_2_‐treated H9C2 cardiomyocytes.

Recently, increasing attention has been focused on autophagy flux, which is the progression of autophagosome formation to cargo delivery and degradation in lysosomes caused by lysosomal proteases.^59^, ^60^ Autophagy flux can be activated by cells to protect against apoptosis and inflammation under stress.^32^, ^61^ It is well known that LC3 and Beclin‐1 are involved in the progression of autophagosome formation, while P62 is an endogenous substrate of the autophagic process, which is considered a marker of autophagic flux, and the accumulation of P62 indicates disrupted autophagy flux.^62^ Our results showed that iPS‐CM enhanced the expression levels of the autophagic proteins LC3 and Beclin‐1, and decreased the accumulation of P62 and the apoptotic protein P53 under oxidative stress. These effects were reversed by LY294002, which is an inhibitor of autophagy flux.^33^ Moreover, exogenous bFGF added to DMEM‐HG activated autophagy flux by up‐regulating the expression of LC3 and Beclin‐1 and down‐regulating the expression of P62 and P53 in H_2_O_2_‐treated H9C2 cardiomyocytes. These results suggested that iPS‐CM acted as a potential agonist of autophagy flux to inhibit the apoptosis of H_2_O_2_‐treated H9C2 cardiomyocytes, which might be partially attributed to the endogenous bFGF. The Wnt/β‐catenin signalling pathway is the classical pathway that regulates cell proliferation.^34^ A closely related report has demonstrated that XAV939, an inhibitor of the Wnt/β‐catenin signalling pathway, counteracted the proliferation of preterm umbilical cord MSCs (UC‐MSCs) compared to term UC‐MSCs.^63^ In this study, we demonstrated that iPS‐CM up‐regulated the levels of p‐β‐catenin (ser 675), β‐catenin (nuclear protein) other than β‐catenin (cytosolic protein) and its target proteins Cyclin D1, c‐Myc as well as Survivin. These effects of iPS‐CM were inhibited by XAV‐933. Furthermore, exogenous bFGF added to DMEM‐HG also up‐regulated the expression of these proteins. These results indicated that iPS‐CM effectively promoted the proliferation of H9C2 cardiomyocytes by activation of the Wnt/β‐catenin signalling pathway, which might be partially attributed to endogenous bFGF.

In conclusion, our study demonstrated that iPS‐CM might partially rely on endogenous bFGF to significantly promote the proliferation of H9C2 cardiomyocytes and inhibit H_2_O_2_‐induced cellular apoptosis. The anti‐apoptotic role of iPS‐CM was correlated with the down‐regulation of the apoptotic proteins BAX and P53 as well as the up‐regulation of the anti‐apoptotic protein BCL‐2. The promotion of the proliferative role of iPS‐CM was involved in the up‐regulation of p‐β‐catenin (ser 675), β‐catenin (nuclear protein) other than β‐catenin (cytosolic protein) and its target proteins Cyclin D1, c‐Myc and Survivin. Furthermore, iPS‐CM inhibited cell senescence and oxidative stress. Additionally, the potential mechanisms including the Wnt/β‐catenin signalling pathway involved in the promotion of H9C2 cardiomyocyte proliferation and the activation of autophagy flux participating in the anti‐apoptotic effects of H_2_O_2_‐treated H9C2 cardiomyocytes after iPS‐CM treatment were revealed. This study might develop an effective way to improve the cardiomyocyte activities, and suggest that iPS‐CM might be a potential therapeutic approach that might benefit patients with myocardial infarction.

## CONFLICTS OF INTEREST

The authors declare no conflict of interest.
